# An RGS4-Mediated Phenotypic Switch of Bronchial Smooth Muscle Cells Promotes Fixed Airway Obstruction in Asthma

**DOI:** 10.1371/journal.pone.0028504

**Published:** 2012-01-12

**Authors:** Gautam Damera, Kirk M. Druey, Philip R. Cooper, Vera P. Krymskaya, Roy J. Soberman, Yassine Amrani, Toshinori Hoshi, Christopher E. Brightling, Reynold A. Panettieri

**Affiliations:** 1 Airways Biology Initiative, Pulmonary, Allergy and Critical Care Division, University of Pennsylvania, Philadelphia, Pennsylvania, United States of America; 2 National Institute of Allergy and Infectious Diseases, National Institutes of Health, Bethesda, Maryland, United States of America; 3 Harvard Medical School, Boston, Massachusetts, United States of America; 4 University of Leicester, Leicester, United Kingdom; 5 Department of Physiology, University of Pennsylvania, Philadelphia, Pennsylvania, United States of America; University of Giessen Lung Center, Germany

## Abstract

In severe asthma, bronchodilator- and steroid-insensitive airflow obstruction develops through unknown mechanisms characterized by increased lung airway smooth muscle (ASM) mass and stiffness. We explored the role of a Regulator of G-protein Signaling protein (RGS4) in the ASM hyperplasia and reduced contractile capacity characteristic of advanced asthma. Using immunocytochemical staining, ASM expression of RGS4 was determined in endobronchial biopsies from healthy subjects and those from subjects with mild, moderate and severe asthma. Cell proliferation assays, agonist-induced calcium mobilization and bronchoconstriction were determined in cultured human ASM cells and in human precision cut lung slices. Using gain- and loss-of-function approaches, the precise role of RGS proteins was determined in stimulating human ASM proliferation and inhibiting bronchoconstriction. RGS4 expression was restricted to a subpopulation of ASM and was specifically upregulated by mitogens, which induced a hyperproliferative and hypocontractile ASM phenotype similar to that observed in recalcitrant asthma. RGS4 expression was markedly increased in bronchial smooth muscle of patients with severe asthma, and expression correlated significantly with reduced pulmonary function. Whereas RGS4 inhibited G protein-coupled receptor (GPCR)-mediated bronchoconstriction, unexpectedly RGS4 was required for PDGF-induced proliferation and sustained activation of PI3K, a mitogenic signaling molecule that regulates ASM proliferation. These studies indicate that increased RGS4 expression promotes a phenotypic switch of ASM, evoking irreversible airway obstruction in subjects with severe asthma.

## Introduction

Asthma, a common respiratory disease, manifests by airway inflammation, hyperresponsiveness and reversible luminal obstruction. Despite research efforts, 15%–25% of patients with asthma develop irreversible airway obstruction, which is refractory to steroids and bronchodilators, and disproportionately account for asthma morbidity and mortality [1], [2]. In biopsies of the bronchial wall, increases in the mass of airway smooth muscle (ASM), the pivotal cell regulating bronchomotor tone, in part defines airway remodeling in severe asthma [2]. However, the contribution of increases in smooth muscle mass to irreversible airway obstruction remains controversial.

Phenotypic plasticity defines the ability of smooth muscle to switch between a contractile and synthetic state [3], [4]. Although characteristic of smooth muscle in cell culture, phenotypic plasticity of smooth muscle in disease states remains controversial [3], [4]. Proliferative smooth muscle manifests abundant organelles for protein and lipid synthesis, increased mitochondria and diminished expression of contractile apparatus and associated proteins [5], [6], [7]. Whether all or select populations of smooth muscle *in vivo* retain a proliferative capacity and whether distinct signaling pathways serve as master switches to promote smooth muscle growth and inhibit agonist-induced contraction remain unknown.

Regulators of G protein signaling (RGS) proteins inhibit GPCR function by binding activated (GTP-bound) Gα subunits and accelerating GTP hydrolysis by Gα [8]. This GTPase accelerating (GAP) activity hastens the return of Gα to an inactive (GDP-bound) form, promoting rapid termination of G protein signaling. Unrelated to their GAP function, RGS proteins of the R4 subfamily, which includes RGS1–5, 8, 10, 13, 18 and 21, regulate activity of PI3K by interacting with its regulatory p85α subunit [9]. The associated PI3K subunit p110 catalyzes the formation of phosphatidylinositol phosphate (PIP)_3_ from membrane PIP_2_, activating signaling molecules involved in cell growth and synthetic function, such as Akt [10]. p85, an adaptor, co-localizes p110 with receptors and signaling complexes at the plasma membrane [11]. RGS13 inhibited rapid antigen-IgE-induced degranulation of mast cells by limiting association of PI3K with the IgE receptor (FcεRI) [9]. In breast cancer cells, RGS16 reduced epidermal growth factor (EGF)-evoked proliferation by inhibiting PI3K signalosome formation [12].

Given the dual function of RGS proteins in GPCR and PI3K signaling and the importance of these pathways for ASM contraction and proliferation, respectively, we investigated their expression and function in human ASM (HASM) cells in severe asthma.

## Materials and Methods

TNFα and IL-1β were obtained from Sigma. PDGF, EGF, thrombin and histamine were sourced from Calbiochem. Signaling inhibitors for PI3K (LY-294002), MEK/ERK (U0126), p38MAPK (SB-203580) and JAK (DBI) were obtained from Calbiochem. RNA extraction and DNAse digestion were performed using RNEasy Kit and RNAse free DNAse from Qiagen. Primers and reagents for RT-PCR were obtained commercially from Applied Biosystems. Ingredients for cell lyse buffers, protease inhibitor cocktails, protein quantification reagents and allied western blotting resources were obtained from BD Biosciences. All cell culture reagents including media and supplements were obtained from nitrogen.

### ASM culture and lentiviral transduction

All *in vitro* studies used HASM isolated from surgically excised tracheal segments of lung tissue donors (NDRI) and cultured in Ham's F-12 medium supplemented with 10% FBS, 100 units/ml penicillin, 0.1 mg/ml streptomycin and 2.5 µg/ml amphotericin B. All experiments were performed using cultures within 2^nd^ to 4^th^ passages, after staining for native contractile proteins using monoclonal antibodies (mAb) against α-smooth muscle actin and myosin (Santa Cruz Biotechnology). Cell purity was consistently over 95%. For development of *ShRGS4* and *ShCnt* cells, ASM cells were transduced by using Polybrene and lentiviral particles encoding RGS4-specific or scrambled non-coding 19–25 nt shRNA sequences (Santa Cruz Biotechnology). After transduction, stable HASM cell lines expressing shRNA were isolated via puromycin selection. After each passage, the efficiency of silencing was confirmed by RT-PCR using gene-specific primers supplied by the manufacturer.

### Clinical subjects

Subjects with asthma and non-asthmatic controls were recruited from Leicester, UK. Subjects with asthma had a consistent history and objective evidence of asthma, as described previously [13]. Subjects underwent extensive clinical characterization including video-assisted fiberoptic bronchoscopic examination. The study was approved by the Leicestershire Ethics Committees. All patients gave their written informed consent.

### Histology and immunofluorescence studies

For staining of bronchial biopsies, sequential 2-µm sections were sectioned from glycomethacrylate-embedded explants and stained using mAb against α-smooth muscle actin (Dako, Ely, UK), and polyclonal RGS4 (Santa Cruz Biotechnology, Heidelberg, Germany) or appropriate isotype controls (Dako). RGS4^+^ cells were enumerated/mm^2^ ASM in the ASM bundle or adjacent to the ASM bundle (<30 µm). A minimum area of 0.1 mm^2^ was considered accessible as described previously [13]. For immunofluorescence experiments to determine PDGF-induced RGS4 expression, near confluent ASM cells on sterile glass coverslips were fixed in 3% paraformaldehyde (Sigma), blocked with 1% BSA and stained with polyclonal RGS4 (N-16, 1∶500), Ab or isotype-matched Ab. Cells were then stained with Alexa Fluor 488-conjugated anti-goat Ab (1∶1,000, Invitrogen) and counterstained with DAPI (4′-6-diamidino-2-phenylindole, Sigma) to identify nuclei.

### Biochemical studies

Co-immunoprecipitation experiments were performed following methodology described earlier [9], [14]. Antibodies against phosphorylated-p85, p85 were obtained from Santa Cruz Biotechnology. For immunoprecipitation experiments, cleared cell lysates were incubated overnight at 4°C with anti-RGS4 tagged TrueBlot anti-goat Ig IP beads (ebiosciences). Protein transfer was followed by overnight incubation with Ab against phosphorylated-p85 (1∶500) and p85 (1∶500). After incubation with HRP-conjugated secondary antibody, the signal was detected with ECL reagent (Promega).

### p-Akt dynamics and Akt kinase assay

Differential kinetics of PDGF-mediated Akt phosphorylation (Ser-473) was assayed in total protein lysates (1 µg/ml) from *ShRGS4* and *ShCnt* using Akt phospho STAR ELISA Kit (Millipore) following enclosed protocols. Akt-dependent phosphorylation of GSK-3 in *ShRGS4* and *ShCnt* cells was determined using an Akt Activity Assay Kit (Abcam).

### Cell counting experiments and cell cycle analysis

*ShCnt* and *ShRGS4* ASM cells were seeded at equal densities (96×10^3^ cells/well) in 6-well plates and cultured for 7 days. Cells were then serum deprived (Ham's F-12 supplemented with 0.1% BSA) for an additional 2 days before treatment with 10 ng/ml PDGF for an additional 72 hours. Cultures were dissociated with 0.5% trypsin-EDTA (Invitrogen) solution and counted in triplicate using the Coulter Z1 cell counter (Beckman Coulter). To analyze cell cycle profiles, *ShCnt* and *ShRGS4* ASM cells treated with/without PDGF were prepared for propidium iodide staining using CycleTest Plus Kit (BD Biosciences). Thereafter, DNA contents of the stained nuclei were analyzed on a FACS Canto flow cytometer and interpreted using Verity ModFit LT 3.0 Software.

### Precision cut lung slices (PCLS) and small airway responses

The smallest lobe of human lungs from lung tissue donors (NDRI) was inflated with 2% (wt/vol) low-melting-point agarose (Sigma), cored (8 mm in diameter) and sliced (250 µm thickness) using a Krumdieck tissue slicer (Alabama Research and Development) as described earlier [15]. Changes in small airway lumen to increasing log concentration of CCh (10^−8^ to 10^−4^ mol/L) in vehicle or PDGF-pre-treated (50 ng/ml for 8 hours) slices were recorded using a CCD camera (Nikon ECLIPSE Model No. TE2000-U, magnification ×40) connected to a live video feed (Evolution QEi, Model No. 32-0074A-130, video recorder). A log half-maximum effective concentration (EC_50_) and maximum drug effect (E_max_) value for each airway were derived from a concentration-response curve.

### Intracellular Ca^2+^ responses

Fura-2-loaded *ShCnt* and *ShRGS4* ASM cells cultured on coverslips were mounted onto an open slide chamber, placed onto an inverted microscope and excited at 340 and 380 nm wavelength, and emissions were collected at ≥450 nm wavelength using a CCD camera (Nikon/Photon Technology International). After subtracting the background signal obtained with Mn^2+^ and ionomycin, the ratio of fluorescence intensities at 340 and 380 nm wavelength was determined using IgorPro (WaveMetrics). The net Ca^2+^ responses to contractile agonists were calculated by subtracting the basal from that of the peak ratio values.

### Statistical analysis

Graph Pad Prizm software was used to determine statistical significance evaluated by a paired Students *t*-test for two groups or analysis of variance (ANOVA) for multiple groups. *P* values of <0.05 were considered significant.

## Results

### Mitogens selectively induce expression of RGS proteins

**Figure 1:**
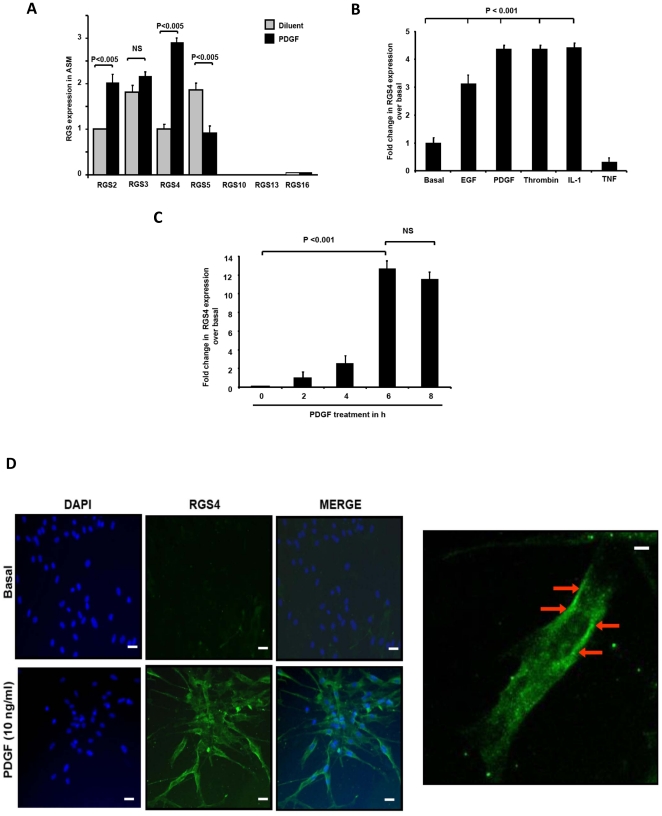
Human airway smooth muscle (HASM) mitogens induce RGS4 expression. (**A**) HASM cells express *RGS* mRNAs. *RGS* expression was determined by real-time PCR in HASM cells left untreated or treated with PDGF for 2 h. Data presented are mean ± SEM of 3 separate experiments performed in triplicate using *Gadph* as an internal control, relative to *RGS2* in untreated cells, set as ‘1’. (**B**) Analysis of *RGS4* expression in HASM cells treated with mitogens such as EGF (1 ng/ml), PDGF (10 ng/ml), thrombin and cytokines by real-time PCR. Data (mean ± SEM) of 5 independent experiments. (**C**) Kinetics of PDGF-mediated *RGS4* mRNA expression as a function of time. Values (mean ± SEM of 3 separate experiments performed in triplicate) are relative to those of untreated cells, set as ‘1’ (**D**) RGS4 expression in untreated (top) or PDGF-treated (bottom) HASM cells immunostained with polyclonal anti-RGS4 followed by Alexa Fluor 488-conjugated anti-IgG and DAPI. Left: High power (650×) visualization of immunostaining with an anti-RGS4 antibody shows plasmalemma staining of RGS4 protein after PDGF. These data are representative of triplicate wells in 2 separate cell lines. Bars represent 50 microns.

Human ASM cells predominantly expressed *RGS2–5* (**Figure 1A**). Because *RGS* transcription often dynamically responds to environmental cues, we analyzed *RGS* expression in HASM treated with growth factors or cytokines associated with airways dysfunction in asthma by real-time PCR. Exposure of HASM to PDGF more effectively induced RGS4 mRNA and protein expression (**Figure 1A**). Increased *RGS4* transcripts were also detected in cells exposed to EGF, thrombin, interleukin-1β (IL-1β) and tumor necrosis factor α (TNFα) compared to untreated cells (**Figure 1B**). PDGF-induced *RGS4* expression was dose- and time-dependent, with maximum expression occurring at 6 hours (**Figure 1C**), and required active transcription as it was inhibited by actinomycin D (**Figure S1A**). PDGF-elicited *RGS4* transcription also required activity of PI3K and ERK1/2 but not p38 mitogen-activated protein (MAP) or Janus (JAK) kinases (**Figure S1B**). Immunofluorescent staining with an RGS4-specific antibody demonstrated minimal RGS4 expression in quiescent cells whereas PDGF induced RGS4 expression in nearly all HASM cells (**Figure 1D**). Consistent with prior studies of other cell types [16], RGS4 localized in the cytoplasm and at the plasma membrane of HASM cells.

### RGS4 expression in ASM increases with asthma disease severity

**Figure 2:**
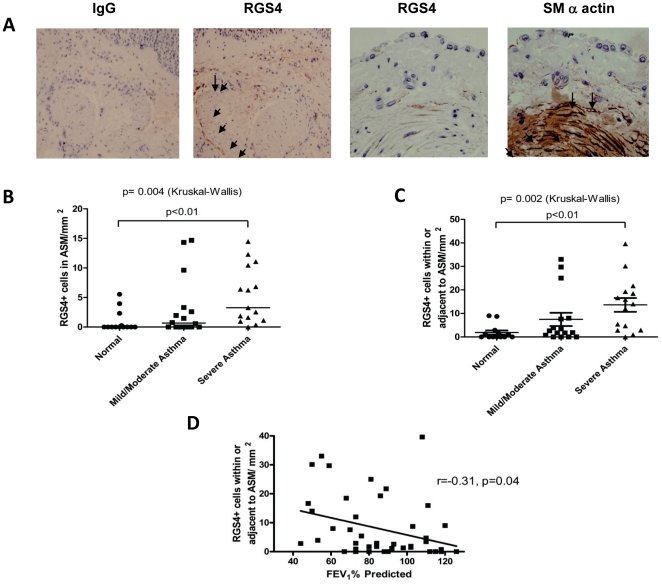
RGS4 is expressed in proportion to lung ASM mass and disease severity in asthma. (**A**) RGS4 cells within and adjacent to the asthma ASM bundle. Representative photomicrographs of a bronchial biopsy from a subject with asthma (200×) stained with isotype control antibody (far left) or anti-RGS4 (3 right panels) which include the epithelium, lamina propria and ASM bundle. Far right panel shows staining of a biopsy from a separate patient (400×) stained with antibodies against α-smooth muscle actin and RGS4. Arrows show RGS4^+^ cells within and adjacent to the ASM bundle. (**B–C**) Dot-plot of the number of RGS4^+^ cells/mm^2^ ASM within the ASM bundle (**B**) or RGS4^+^ cells/mm^2^ ASM within and adjacent to the ASM bundle (**C**) in subjects with asthma and healthy controls. Horizontal bars depict the median value (*P*<0.05, Kruskal-Wallis test for all across-group comparisons; *P* value for Dunn's post test given on figure). (**D**) Correlation of RGS4^+^ cells/mm^2^ ASM with % predicted FEV_1_ in all subjects and those with asthma alone, respectively, with correlation coefficient and *P* value provided.

The selective induction of RGS4 by HASM mitogens suggested a potential function in the ASM hyperplasia and fixed airway obstruction associated with severe asthma. To characterize RGS4 expression in bronchial ASM bundles, we evaluated endobronchial biopsies from patients with asthma and age-matched healthy controls by immunohistochemistry. We categorized patients into 3 groups according to criteria established by the Global Initiative for Asthma (GINA, http://www.ginasthma.com): healthy, mild-moderate asthma and severe asthma (**Table 1**). Smooth muscle bundles in bronchi from those with asthma had markedly increased numbers of RGS4^+^ ASM cells compared to those with mild-moderate asthma or healthy subjects (**Figures 2A–C**). RGS4^+^ cells were found at the periphery of the ASM bundle although some cells within the bundles also stained positively for α-smooth muscle actin and RGS4. Notably, the number of RGS4^+^ cells correlated inversely with pulmonary function as assessed by the forced expiratory volume in 1 second (FEV_1_) (**Figure 2D**).

**Table 1:** Clinical and sputum characteristics of patients categorized according to GINA.

	Normal	Mild-Moderate Asthma (GINA 1 = 11, 2 = 3, 3 = 2)	Severe Asthma (GINA 4 = 11, 5 = 4)
Number	13	16	15
Age*	47 (4)	49 (4)	52 (3)
Male/Female	8/5	6/9	5/10
Never/current/ex-smokers	11/0/2	12/0/4	12/0/3
Pack years*	0.5 (0.4)	2.9 (1.5)	1.5 (0.9)
Atopy n (%)	7 (54)	11 (69)	10 (67)
PC_20_FEV_1_ (mg/ml)†	>16	0.37 (0.14–0.97)**	1.1 (0.3–3.6)**
FEV_1_% predicted*	97 (4)	83 (6)	79 (7)**
Pre-BD FEV_1_/FVC %*	83 (2)	72 (2)	68 (4)
BD response (%)*	X	12 (4)	9 (3)
Inhaled corticosteroids (BDP/day)	0	300 (139)	1645 (197)
Oral corticosteroids n (%)	0	0	4 (27)
LABA n (%)	0 (0)	2 (13)	15 (100)
Sputum Cell Counts			
TCC*	1.2 (0.2)	2.4 (0.5)	4.7 (1.2)**
Eosinophils %‡	0.3 (0.8)	1.0 (5.7)	2.8 (48)
Neutrophils %*	47 (13)	51 (8)	58 (8)
Macrophages %*	51 (9)	38 (6)	25 (6)**
Lymphocytes %*	1.8 (0.8)	1.0 (0.2)	1.5 (0.7)
Epithelial cells %*	3 (2)	4 (1)	7 (3)

GINA: Global Initiative for Asthma; BD: bronchodilator; LABA: long-acting bronchodilator; FEV_1_: forced expiratory volume in one second; TCC: total cell counts; BDP: beclomethasone dipropionate; PC_20_FEV_1_: provocative concentration of methacholine to induce a 20% decrease in FEV1;

*mean (SE);

† geometric mean (95% CI);

‡ median (IQR);

**P<0.05 compared to control.

### RGS4 expression is required for mitogen-induced myocyte proliferation

**Figure 3:**
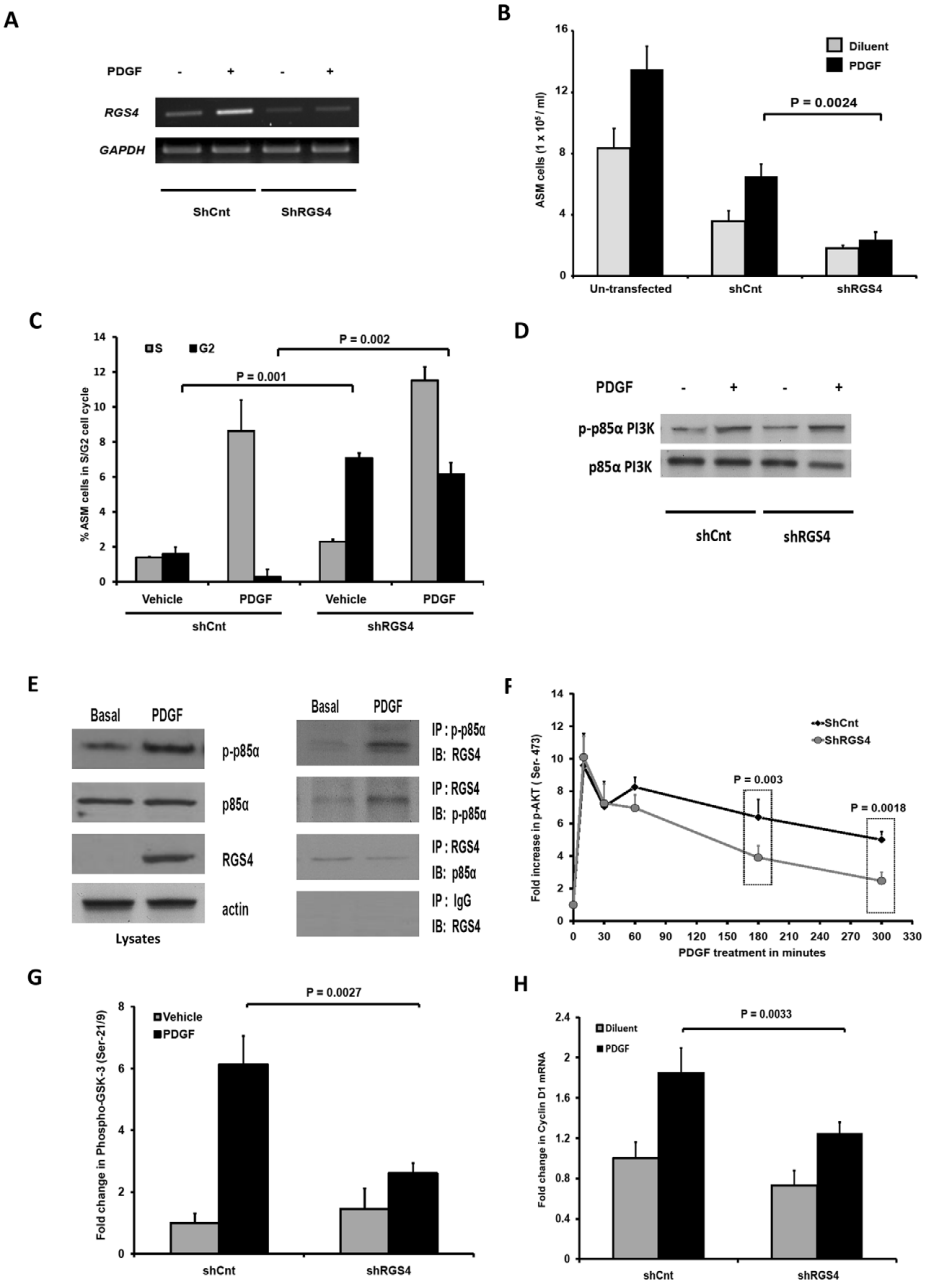
RGS4 is required for PI3K and Akt-dependent HASM proliferation. (**A**) Gel photographs of RT-PCR analysis of RGS4 and GAPDH expression in untreated and PDGF-treated HASM cells infected with lentiviruses encoding either control (*ShCnt*) or RGS4-specific (*ShRGS4*) shRNAs. (**B**) RGS4 depletion attenuates PDGF-mediated HASM proliferation. Untransfected *ShRGS4* and *ShCnt* ASM cells were serum-starved for 24 h followed by treatment with serum-free medium or medium containing PDGF for an additional 72 h. Total cell numbers were determined using a Beckman Cell Coulter counter (mean ± SEM of 6 separate experiments performed in 2 cell lines). (**C**) PDGF-induced cell cycle traversal after 24 h analyzed by FACS analysis of propidium iodide-stained nuclei isolated from *ShRGS4* and *ShCnt* HASM cells. (**D**) Analysis of p85α phosphorylation assessed by immunoblotting of lysates of untreated or PDGF-treated S*hCnt* or *ShRGS4* expressing HASM cells. Blots are representative of 3 separate experiments performed in 2 cell lines. (**E**) Left: Immunoblot analysis of p-p85 PI3K, RGS4 and total p85 PI3K expression in untreated or PDGF-treated cells. Right: Immunoblot analysis of lysates from untreated or PDGF-treated cells immunoprecipitated with indicated antibodies. Blots are representative of 3 separate experiments performed in 3 cell lines. (**F**) Kinetics of PDGF-mediated Akt phosphorylation were analyzed in lysates of *ShRGS4* or *ShCnt* HASM cells by ELISA. Data (mean ± SEM) are expressed as fold change over basal, set as ‘1’ in 5 separate experiments. (**G**) Akt kinase activity in *ShRGS4* or *ShCnt* HASM cells in untreated or PDGF-treated cells. p-Akt (Ser-473) was immunoprecipitated from total cell lysates using a specific antibody followed by incubation with recombinant GSK3. Phospho-GSK-3α/β (Ser-21/9) was quantified by colorimetric assay. Data (mean ± SEM) are fold-change over vehicle-treated *ShCnt* cells determined in 4 independent experiments measured in triplicate. (**H**) Total RNA was extracted from *ShRGS4* and *ShCnt* HASM cells treated with PDGF or diluent for 8 h followed by analysis of relative *cyclin D1* expression by real-time PCR. Data are mean ± SEM of 6 independent experiments using 2 cell lines.

Since RGS4 expression increased in proportion to ASM mass in asthma and correlated with the severity of disease, we next addressed whether ASM proliferation requires RGS4 expression. To test this directly, we extinguished RGS4 expression in cultured HASM cells using siRNA and measured proliferation in the presence and absence of PDGF. RGS4 amounts were reduced 64±6% in cells expressing an RGS4-specific shRNA relative to cells expressing a scrambled control shRNA (**Figure 3A**). Unexpectedly, PDGF-evoked proliferation was profoundly reduced in RGS4-depleted cells compared to control in the presence or absence of PDGF (**Figure 3B**). RGS4 deficiency led to a 20-fold increase in the percentage of cells in G_2_ phase of the cell cycle compared to control, indicating growth arrest (*P*<0.0005) (**Figure 3C**). To exclude the possibility that reduced cell numbers were due to apoptosis or necrosis, we measured caspase 3 activity or LDH levels, respectively. While ceramide treatment induced HASM apoptosis as evidenced by a 10-fold increase in caspase 3 activity, minimal caspase 3 activity was detected in control or RGS4-deficient cells in the presence or absence of PDGF (**Figure S2A**). Accordingly, assessment of LDH levels showed no significant differences in cell viability across all study groups (**Figure S2B**).

These results suggest that, in contrast to the inhibition of PI3K-mediated proliferation of neoplastic cells by RGS16, mitogen-induced cell cycle progression of primary HASM cells unexpectedly *required* RGS4. Among several critical signaling events, robust and durable activation of PI3K and its downstream effector S6K1 is required for HASM mitogenesis [1], [17]. We hypothesized that RGS4 mediates PDGF-elicited HASM growth by interacting with the phosphorylated p85α subunit of PI3K and modulating activity of the PI3K signaling pathway. PDGF induced tyrosine phosphorylation in HASM cells (**Figure 3D**). Consistent with our previous studies with mast cells [9], we observed equivalent p85α phosphorylation in control and RGS4-depleted HASM cells. To characterize RGS4-p85α interactions in HASM, we immunoprecipitated proteins from untreated or PDGF-treated cells. We detected RGS4 specifically in immunoprecipitates of phospho-p85α from PDGF-treated but not untreated cells (**Figure 3E**). Similarly, immunoprecipitation of RGS4 from lysates of PDGF-treated HASM cells also extracted p-p85α PI3K.

To determine the molecular consequence(s) of RGS4-phospho-p85α binding for PI3K signaling in HASM cells, we analyzed Akt phosphorylation quantitatively by enzyme-linked immunosorbent assay (ELISA). Growth factors including PDGF elicit Akt phosphorylation at Thr-308 and Ser-473 residues, resulting in phosphorylation of proliferation-critical effectors such as GSK-3 and S6K1 [18]. PDGF induced rapid Akt phosphorylation in HASM cells as early as 5 minutes after stimulation, which persisted for up to 5 hours (**Figure 3F**). Although we detected similar levels of phospho-Akt in control and RGS4-shRNA expressing cells at early time points, Akt phosphorylation was dramatically reduced in RGS4-depleted cells 1–5 hours following PDGF treatment. Consistent with the importance of Akt phosphorylation for its kinase activity, prolonged PDGF treatment of HASM elicited Akt activation as assessed by substrate (GSK-3β phosphorylation by Akt immunoprecipitated from PDGF-treated cells) (**Figure 3G**). We observed substantially reduced Akt kinase activity in cells expressing RGS4 shRNA compared to control. Finally, to determine the requirement of RGS4 for PDGF-induced PI3K- and Akt-dependent cell cycle progression, we evaluated expression of the Akt target gene *cyclin D1* by quantitative real-time PCR [19], [20], [21]. As expected, RGS4 depletion decreased PDGF-evoked *cyclin D1* expression (**Figure 3H**). The requirement of RGS4 for mitogen-induced cell growth was specific to the PI3K pathway, as siRNA-mediated extinction of RGS4 had no effect on PDGF-evoked ERK phosphorylation in HASM (**Figure S3**). Collectively, these studies demonstrate that PDGF induction of RGS4 expression has a critical function in mitogen-induced ASM hyperplasia through regulation of the PI3K-Akt signaling axis.

### Mitogen-induced expression of RGS4 attenuates ASM excitation-contraction coupling

**Figure 4:**
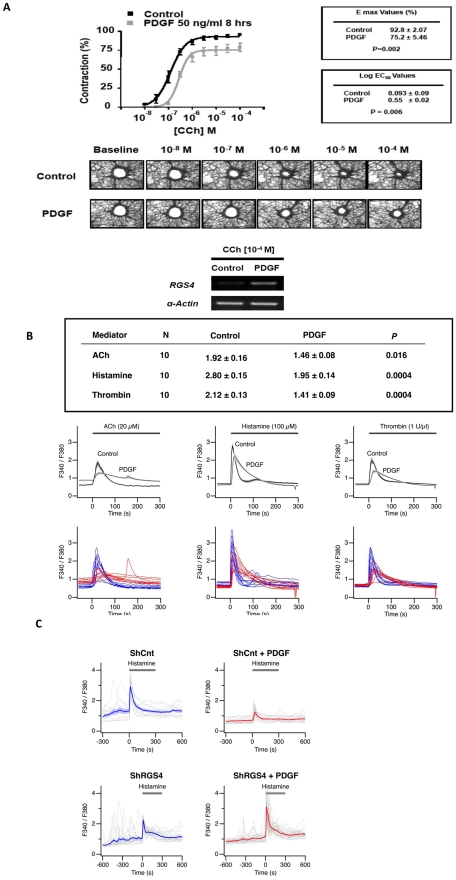
PDGF markedly inhibits carbachol-induced bronchoconstriction in human small airways. (**A**) PCLS were obtained from healthy donors and treated with medium or PDGF (50 ng/ml) for 8 h followed by analysis of carbachol-induced small airway constriction by microscopy. Log EC_50_ and E_max_ values (mean ± SEM) were determined in experiments on 16 airways obtained from 4 separate donors as described in Methods. (**B**) PDGF attenuates agonist-induced increases in [Ca^2+^]_i_. HASM cells were stimulated for 8 h with 10 ng/ml PDGF or diluent followed by measurement of [Ca^2+^]_i_ using a Ca^2+^-sensing fluorophore after stimulation with acetylcholine (ACh), histamine or thrombin. Single-cell calcium transients were measured over a period of 300 sec. Table shows mean ± SEM of peak [Ca^2+^]_i_ levels determined in 30 cells (*P* values determined by 2-tailed Student's *t* test). Bottom tracings represent [Ca^2+^]_i_ in individual cells (blue line = control, diluent-treated; red line = PDGF-treated). Middle tracings represent group mean data ± SEM shown in the shaded segments. (**C**) RGS4 is required for PDGF-mediated attenuation of agonist-induced [Ca^2+^]_i_ in HASM cells. The mean responses are shown using thick curves, and the individual cell responses are shown using dashed curves.

Since RGS4 expression is upregulated in failing human hearts [22], and overexpression of RGS4 impairs cardiac myocyte contractility and increases heart failure in a mouse model [23], we next examined whether mitogens attenuate agonist-induced contractile responses in an RGS4-dependent manner. GPCR agonists including carbachol, thrombin and histamine induce ASM contraction through a Gαq-dependent pathway that increases intracellular Ca^2+^ concentrations. We treated human precision cut lung slices (PCLS) with PDGF and measured airway contraction by supravital microscopy. PDGF dramatically upregulated *RGS4* expression in lung slices (**Figure 4A**). Compared to untreated slices, PDGF reduced the potency (log EC_50_) of carbachol, an agonist of the M3 muscarinic receptor, as well as maximal carbachol-evoked bronchoconstriction (E_max_) (**Figure 4A**). PDGF also diminished histamine-induced contraction, suggesting that PDGF modulates contractile responses downstream of receptor activation through RGS4 upregulation.

To test this directly, we treated cultured HASM cells with PDGF and measured intracellular Ca^2+^ flux in quiescent and agonist-treated cells. Acetylcholine (M3 receptor agonist), histamine or thrombin induced a rapid increase in intracellular Ca^2+^ levels, and PDGF pre-treatment markedly blunted these responses (**Figure 4B**). In approximately 15% of HASM cells, PDGF rendered cells completely unresponsive to agonists. To determine whether PDGF inhibition of bronchoconstriction required RGS4, we compared agonist-induced Ca^2+^ responses in control and RGS4-depleted cells. Knockdown of RGS4 completely reversed PDGF-induced inhibition of histamine-evoked Ca^2+^ flux (**Figure 4C**). In parallel, we also determined that PDGF had little effect on isoproterenol-induced bronchodilation of carbachol-stimulated bronchoconstriction as shown in **Figure S4**.

## Discussion

Severe asthma encompasses a variety of phenotypes that are characterized by ages of onset [24], duration of disease, degree of airflow impairment, presence of co-morbidity and types of inflammation [25], [26]. The majority of subjects with severe asthma manifest a degree of irreversible airway obstruction despite maximal bronchodilation and, in some, a lack of methacholine responsiveness [25], [27], [28]. Severe asthma patients also experience more frequent and sustained exacerbations as compared with that of mild/moderate patients [24], [25], [26], [27], [28]. Given the irreversible component of the disease, investigators have suggested in part that airway remodeling and ASM hyperplasia may contribute to fixed airway obstruction [24], [26], [27], [28]. We now show that human ASM proliferation requires expression of RGS4 protein, which interacts with the p85 subunit of PI3K. RGS4 also inhibits agonist-induced bronchoconstriction and calcium mobilization. Further, RGS4 expression in ASM cells is associated with increasing disease severity and may serve as a unique biomarker and/or therapeutic target to abrogate ASM hyperplasia and irreversible airway obstruction in asthma.

The most well-known function of RGS proteins is to reduce signaling output from GPCR activation. The importance of RGS proteins in the dynamic control of signaling is supported by changes in mRNA for these proteins under a variety of conditions [14], [29]. Different RGS proteins interact with varying preference with members of the G_i/o_ and/or G_q_ families to reduce signaling. In addition to the RGS domain, RGS proteins have a variety of domains for non-GPCR protein-protein interactions, and thus selectivity for activation of particular pathways may be obtained by scaffolding mechanisms. Although few investigators have explored whether RGS proteins modulate RTK signaling pathways [30], [31], [32], [33], our data suggest that RGS4 is essential to regulate RTK-mediated ASM growth. RGS expression is highly tissue- and cell-specific and, as such, imparts unique control of cellular function [29], [34].

However, although the RGS family includes GPCR kinases (GRKs) such as β-adrenergic receptor kinase, about which much is known, the function of the smallest RGS molecules, namely, the B/R4 subfamily (RGS1–5, 8, 13, 16, 18, 21), remains unclear. In HASM, we show that the B/R4 RGS3, 4 and 5 proteins are the dominant RGS molecules expressed. Since RGS proteins can profoundly modulate GPCR signaling downstream from the receptor, conceptually B/R4 RGS proteins may modulate agonist-induced ASM contractility at multiple levels. In aortic smooth muscle, knockdown of RGS3 increased muscarinic 3 receptor (M3R)-dependent ERK activation but had no effect on angiotensin II-evoked signaling, suggesting receptor selectivity [35], [36].

Given the necessity of RGS4 in regulating ASM proliferation, we posited that patients with severe asthma, who manifested marked increases in bronchial smooth muscle mass and irreversible airway obstruction, would manifest increased numbers of RGS4^+^ ASM cells. The numbers of RGS4^+^ ASM cells correlated with increasing disease severity. Interestingly, the RGS4^+^ myocytes localized in discrete areas of the bronchial smooth muscle bundle suggesting heterogeneity of expression among myocytes. The recent development of bronchial thermoplasty, which delivers a thermal injury to the bronchial wall, appears to decrease ASM mass and improve clinical outcomes in severe asthma [37], [38]. The location of the RGS4^+^ ASM cells and the requirement of RGS expression in mediating ASM proliferation suggest a particular susceptibility of these cells to such an injury. Since the ASM cells fail to regenerate after the thermal injury, RGS4^+^ cells may represent a unique population of ASM akin to skeletal myoblasts that serve to replenish differentiated muscle.

Although phenotypic plasticity of smooth muscle has been recognized for decades, the precise signaling pathways that inhibit contractile responses and that promote smooth muscle growth remain unknown. In cultured vascular smooth muscle, mostly RGS2 expression, but also 1, 3 and 4, are increased after stimulation with angiotensin II, PDGF, IL-1β or TGFβ [30], [31]. RGS5 is expressed in rat and human aortic smooth muscle but not coronary or venous myocytes [35]. In cardiac muscle, RGS4 plays a critical role in regulating the chronotropic actions of acetylcholine. Lack of RGS4 enhances sensitivity to carbachol-induced bradycardia and evokes arrhythmias [39]. Others reported that increases in cardiac muscle expression of RGS4 decreases cardiac inotropy that promotes heart failure [22]. In myometrial smooth muscle, RGS12 expression is markedly increased at term while other RGS proteins are unchanged [40], [41]. We now show that RGS4 promotes mitogen-induced ASM growth through regulation of PI3K activity yet inhibits agonist-induced contractile function by decreasing calcium mobilization stimulated by agonists. Importantly, PDGF had little effect on β-agonist-induced bronchodilation. Increases in RGS4 expression by growth factors, which enhance ASM mitogenesis, may impart a susceptibility to ASM hyperplasia in asthma. In our study, cultured ASM cells were derived from the proximal airway while the contractile responses were measured in the distal airway. To address whether the proximal and distal ASM responded differentially to agonists, cytosolic calcium mobilization to agonists was also studied in the cultured ASM and demonstrated that PDGF modulated agonist-induced calcium levels in the cultured ASM. Despite these studies, we recognize that proximal and distal ASM *in vivo* may undergo differential growth responses, and further experiments are needed to definitively demonstrate whether growth factors modulate RGS4 expression, contraction and proliferation *in vivo*.

**Figure 5:**
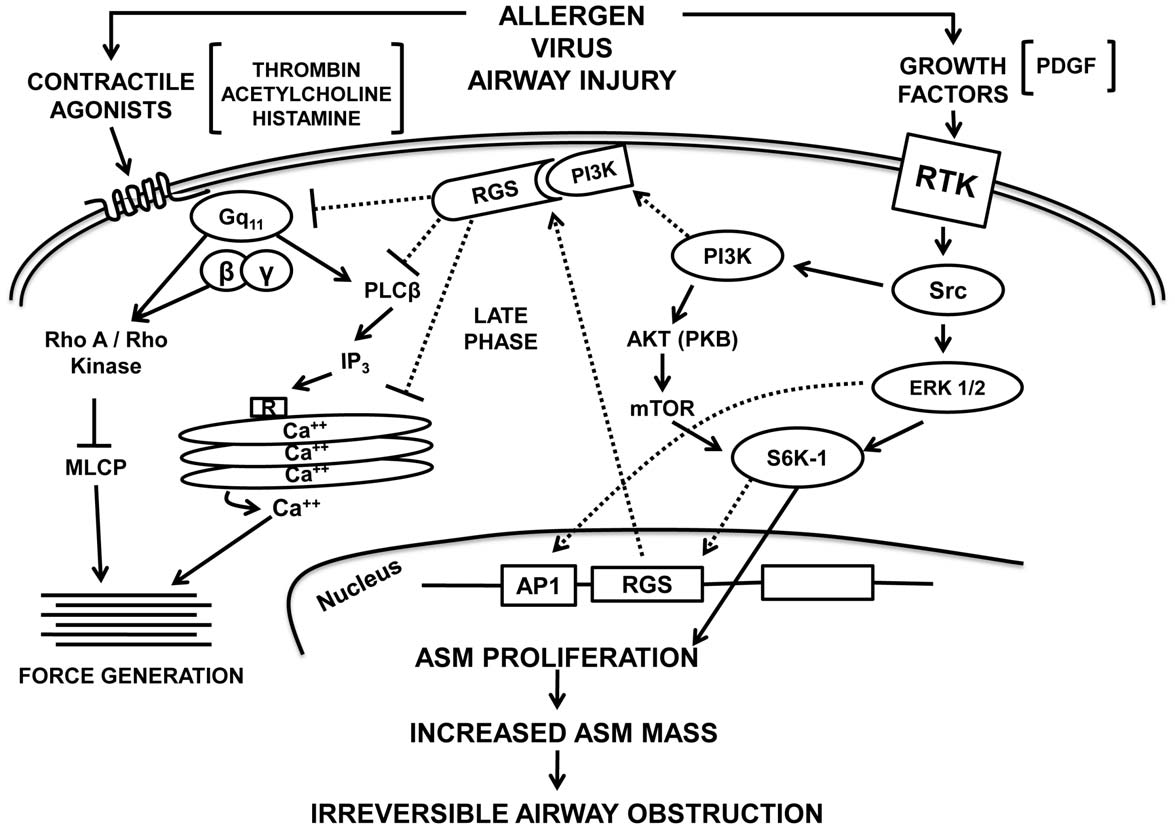
A schematic illustration of the role of RGS4 in modulating human ASM excitation-contraction coupling and mitogen-induced proliferation. A model by which regulatory G protein signaling (RGS) molecules modulate agonist-induced contraction and growth factor-induced mitogenesis. Growth factors stimulate receptor tyrosine kinases (RTK) coupled to small G proteins such as Ras and Src. Src then activates phosphoinositide 3-kinase type IA (PI3K) and extracellular receptor kinase 1/2 (ERK1/2). Subsequently, PI3K activates protein kinase B (PKB) and mammalian target of rapamycin (mTOR) that then stimulates S6 kinase (S6K1) phosphorylation. S6K1 phosphorylation induces expression of RGS and other proteins necessary for cell cycle progression and proliferation. The expression of RGS colocalizes with PI3K and promotes prolonged PI3K activation to facilitate cell cycle traversal. Agonists stimulate G protein-coupled receptors (GPCRs) through the activation of specific Gα subunits. PLCβ stimulation then generates IP_3_ that mediates calcium release through cytoplasmic calcium stores by binding to the IP_3_ and ryanodine receptors. Increased calcium promotes actin-myosin cross bridge cycling in a myosin light chain (MLC) kinase-dependent manner. In parallel, G protein activation stimulates Rho kinase activation that inhibits MLC phosphatase and also promotes regulatory MLC phosphorylation (calcium sensitivity). Agonists and growth factors may activate ASM in a paracrine or autocrine manner. The activation of RTK pathways and the inhibition of agonist-mediated force generation promote ASM hyperplasia that increases ASM mass and may contribute to irreversible airflow obstruction.

Although asthma is considered a disease of reversible airway obstruction and inflammation, patients with severe disease experience irreversible airflow obstruction refractory to current therapies. Given the strikingly increased morbidity seen in this subset of patients compared to those with mild/moderate asthma, the need for new therapeutic approaches remains dire. We have identified growth factor-mediated upregulation of RGS4 in ASM as a deleterious event in severe asthma. RGS4 was required for ASM hyperplasia and rendered cells poorly contractile, consistent with a maladaptive phenotypic switch as shown in **Figure 5**. Although asthma is characterized by airway hyperresponsiveness, the data herein suggest that in severe disease, ASM becomes less responsive, which fixes the airway luminal diameter. Therapeutic approaches that decrease RGS4 expression or antagonize RGS4 function may prevent ASM hyperplasia and irreversible airway obstruction while promoting a more responsive smooth muscle phenotype.

## Supporting Information

Figure S1 **Mitogens induce RGS4 expression that requires PI3K and ERK activation.** (**A**) Mitogens transcriptionally induce RGS4 in ASM cells. Pre-treatment of ASM cells with actinomycin (5 µM) for 1 h abrogated PDGF-, EGF- and thrombin-mediated RGS4 enhancement. (**B**) Assessment of signaling mechanisms mediating PDGF-induced RGS4 transcription. Real-time PCR analysis of HASM cells pre-treated with pharmacological inhibitors of PI3K (10 µM), ERK (10 µM), p38 MAPK (10 µM) or JAK (100 nM) for 1 h prior to treatment with PDGF for an additional 6 h. Data are mean ± SEM of 4 separate experiments performed in triplicate.
(TIF)

Figure S2 **Inhibition of ASM proliferation by silencing RGS4 has little effect on ASM apoptosis.** (**A**) Apoptosis as assessed by caspase 3 activity in PDGF or diluent-treated untransfected (Untfr) *shCnt-* or *shRGS4*-expressing HASM cells after 72 h. As a positive control, ceramide (40 µM) was used as an inducer of caspase-dependent apoptosis. (**B**) LDH levels as a measure of cell viability in Untfr *shCnt* or *shRGS4* HASM cells. As a positive control, Triton X-100 (3%) was used as an inducer of cell toxicity. Data are mean ± SEM of 4 separate experiments performed in triplicate. Values (mean ± SEM of 3 separate experiments performed in triplicate) are relative to those of vehicle-treated Untfr cells, set as ‘1’.
(TIF)

Figure S3 **RGS4 depletion has no effect on PDGF-induced ERK phosphorylation.** Total cell lysates from PDGF- or diluent-treated *shCnt-* or *shRGS4*-expressing HASM cells were immunoblotted by using p-ERK1/2. Total ERK1/2 expression was used as a control for protein loading. Blots are representative of 3 separate experiments performed in 2 cell lines.
(TIF)

Figure S4 **PDGF has little effect on isoproterenol-induced bronchodilation.** PCLS were treated for 8 h with PDGF (50 ng/ml), then bronchoconstricted with carbachol, and cumulative additions of isoproterenol then added. As shown, PDGF had little effect on isoproterenol-induced bronchodilation. These experiments were performed in 5 slices obtained from 3 donors, and the data represent mean ± standard deviations.
(TIF)
