# Supplementary figures and images for: An RGS4-Mediated Phenotypic Switch of Bronchial Smooth Muscle Cells Promotes Fixed Airway Obstruction in Asthma
Source: PLoS One. 2012 Jan 12;7(1):e28504. doi: 10.1371/journal.pone.0028504 (PMC3257220; doi:10.1371/journal.pone.0028504)

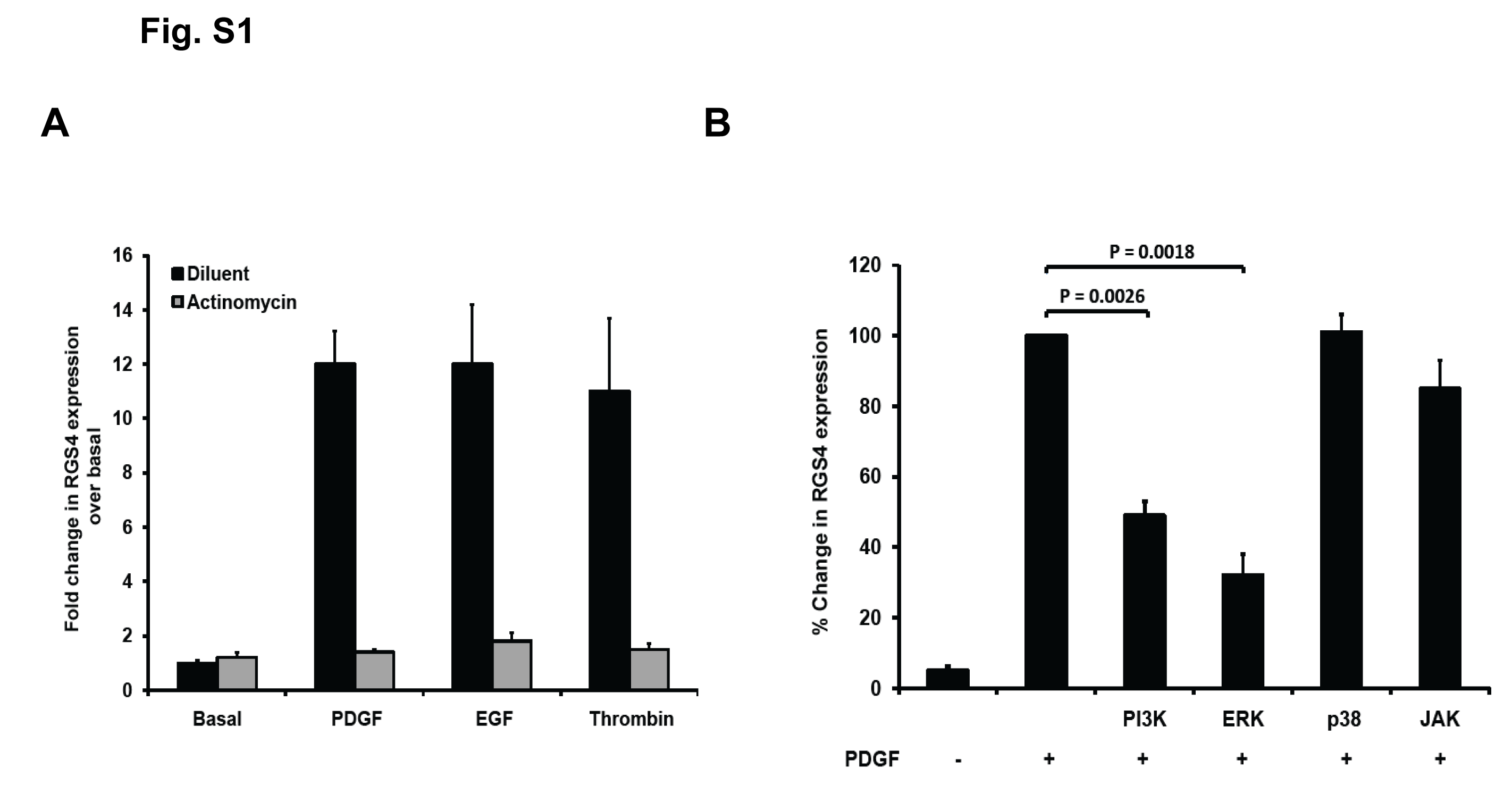

Supplement: Figure S1 — Mitogens induce RGS4 expression that requires PI3K and ERK activation. (A) Mitogens transcriptionally induce RGS4 in ASM cells. Pre-treatment of ASM cells with actinomycin (5 µM) for 1 h abrogated PDGF-, EGF- and thrombin-mediated RGS4 enhancement. (B) Assessment of signaling mechanisms mediating PDGF-induced RGS4 transcription. Real-time PCR analysis of HASM cells pre-treated with pharmacological inhibitors of PI3K (10 µM), ERK (10 µM), p38 MAPK (10 µM) or JAK (100 nM) for 1 h prior to treatment with PDGF for an additional 6 h. Data are mean ± SEM of 4 separate experiments performed in triplicate. (TIF) [file pone.0028504.s001.tif]

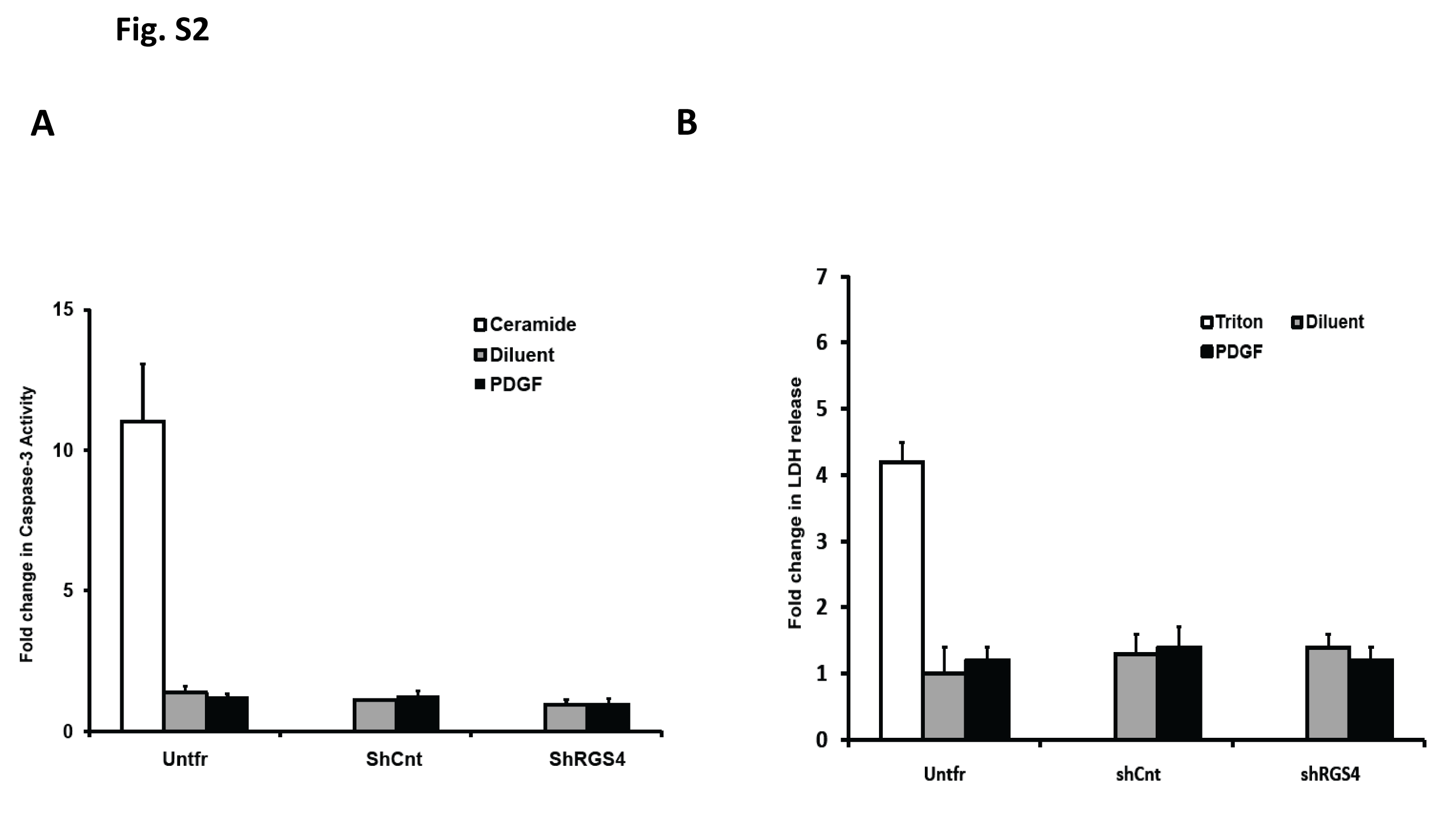

Supplement: Figure S2 — Inhibition of ASM proliferation by silencing RGS4 has little effect on ASM apoptosis. (A) Apoptosis as assessed by caspase 3 activity in PDGF or diluent-treated untransfected (Untfr) shCnt- or shRGS4-expressing HASM cells after 72 h. As a positive control, ceramide (40 µM) was used as an inducer of caspase-dependent apoptosis. (B) LDH levels as a measure of cell viability in Untfr shCnt or shRGS4 HASM cells. As a positive control, Triton X-100 (3%) was used as an inducer of cell toxicity. Data are mean ± SEM of 4 separate experiments performed in triplicate. Values (mean ± SEM of 3 separate experiments performed in triplicate) are relative to those of vehicle-treated Untfr cells, set as ‘1’. (TIF) [file pone.0028504.s002.tif]

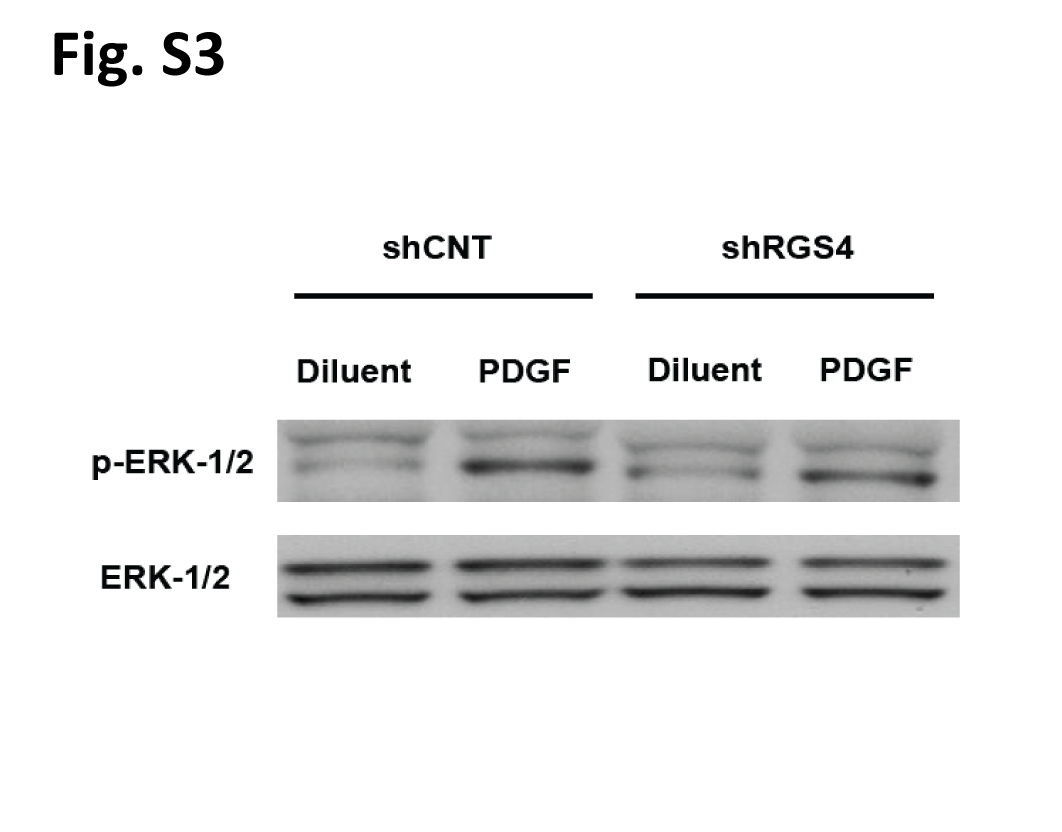

Supplement: Figure S3 — RGS4 depletion has no effect on PDGF-induced ERK phosphorylation. Total cell lysates from PDGF- or diluent-treated shCnt- or shRGS4-expressing HASM cells were immunoblotted by using p-ERK1/2. Total ERK1/2 expression was used as a control for protein loading. Blots are representative of 3 separate experiments performed in 2 cell lines. (TIF) [file pone.0028504.s003.tif]

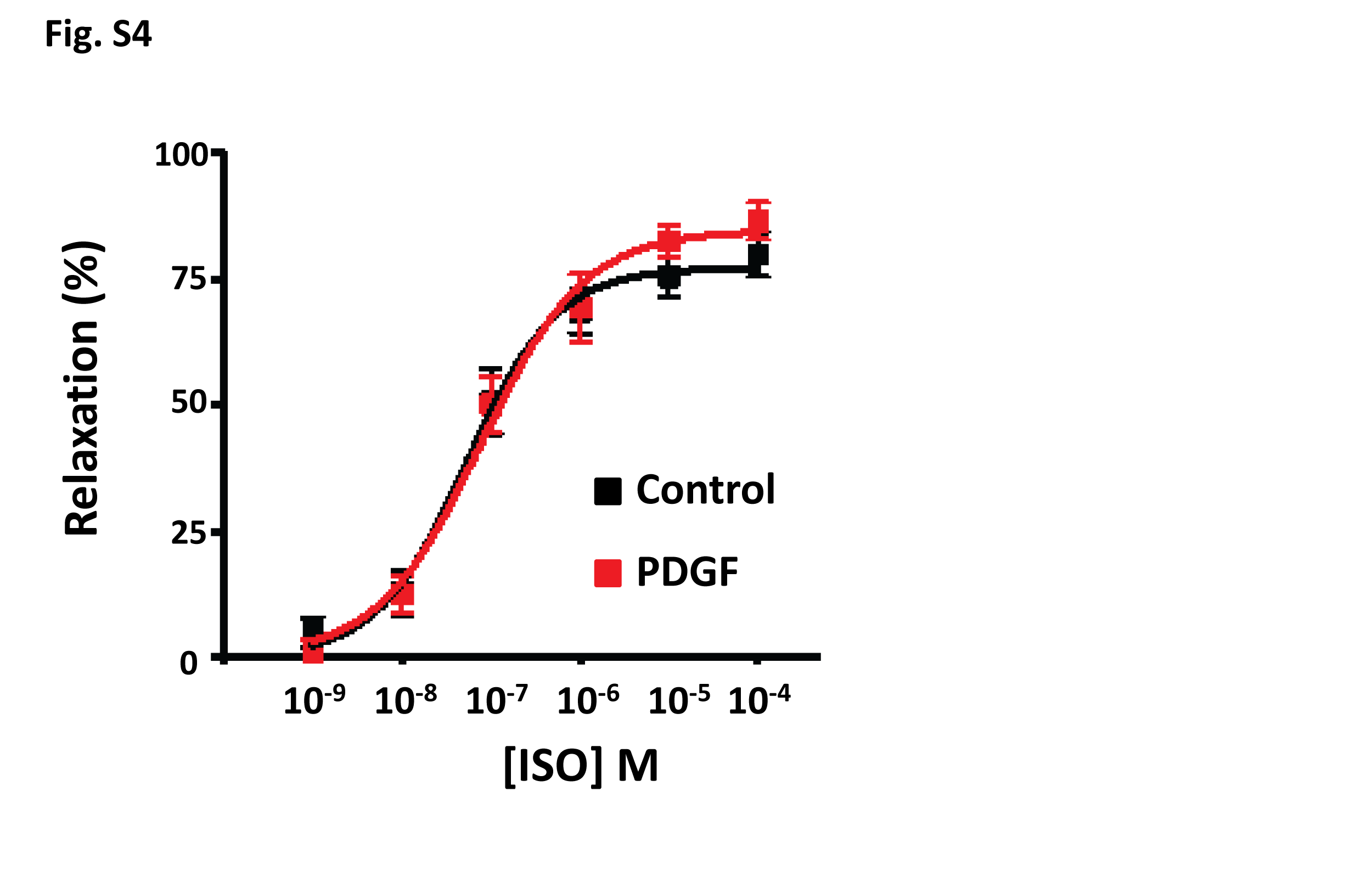

Supplement: Figure S4 — PDGF has little effect on isoproterenol-induced bronchodilation. PCLS were treated for 8 h with PDGF (50 ng/ml), then bronchoconstricted with carbachol, and cumulative additions of isoproterenol then added. As shown, PDGF had little effect on isoproterenol-induced bronchodilation. These experiments were performed in 5 slices obtained from 3 donors, and the data represent mean ± standard deviations. (TIF) [file pone.0028504.s004.tif]
